# Criteria for the qualification of geriatric patients to the trauma center

**DOI:** 10.1186/s13017-026-00683-1

**Published:** 2026-03-08

**Authors:** Małgorzata Sulej-Niemiec, Magdalena Żurowska-Wolak, Andrzej Kopta, Łukasz Kręglicki, Mirosław Szura

**Affiliations:** 1 Department of Emergency Medical Services, Faculty of Health Sciences, Jagiellonian University Medical College, Michałowskiego St. 12, 31-126 Cracow, Poland; 2 Clinic of Surgery, Faculty of Health Sciences, Jagiellonian University Medical College, Cracow, Poland

**Keywords:** Geriatric age, Geriatric trauma, Elderly trauma, Severe injuries, Trauma center, Trauma qualification criteria, Triage criteria, Geriatric triage criteria, Trauma triage, Injury assessment

## Abstract

**Background:**

The growth of the geriatric population, resulting in an exponential increase in the number of injured older people, determines the necessity of ensuring adequate access to specialized units such as trauma centers (TCs). The specificity of pathophysiological processes that progress with age, worsening the body's response to trauma, makes it difficult to develop an optimal triage protocol for geriatric patients, reducing high undertriage, i.e., underestimation of injuries and referral to lower-reference units.

**Aim:**

To verify the TC qualification criteria for 65+ patients and analyze the weights of individual triage factors for these patients.

**Methods:**

This was a retrospective analysis of the medical records of ED patients from the Clinical Hospital with TC in Poland admitted from 1.01.2017 to 31.12.2020. Among 155,320 ED patients, 6541 who formed Trauma group 65+ were selected, in which the TRISS score was estimated. The TC 65+ group meeting the currently applicable TC qualification criteria was selected. The TRISS cutoff value, TRISS 65+ group and TC-omitted 65+ group were determined. The significance of TC qualification factors was estimated. Receiver operating characteristic (ROC curve) analysis and multiple linear regression analysis were used for statistical classification.

**Results:**

The value of ≤ 88.84% determined the threshold for TC qualification, and the leakage of criteria was 58.51%. Six significant factors with different typing weights were identified: GCS ≤ 8 (− 42.90%), extensive crush injury of the extremities (− 33.74%), and RR < 10/min (− 16.16%), blunt injury to thoracic internal organs (− 13.75%), pelvic fracture (− 11.35%) and SBP ≤ 80 mmHg (− 10.41%) were performed. The weight + SD of each factor reduced the potential TRISSe value to ≤ 88.84% (threshold). Modifications of the cutoff values of significant physiological parameters were determined, i.e., GCS ≤ 14 (sensitivity = 79.79%, specificity = 98.25%, AUC = 0.896, Youden index = 0.780) and SBP ≤ 129 mmHg with questionable efficacy of the result.

**Conclusions:**

The current TC qualification criteria require modification for geriatric patients, which would complete the leakage estimated at 58.51% according to the TRISS scale. The leakage results from underestimation of the weight of the 6 triage factors to the TC, and the modification of the criteria should include a reduction in the current requirement of 4 factors to 1 and allow admission to the TC of a 65+ trauma patient with one of the following anatomical injuries: extensive crush injury of extremities, blunt injuries with symptoms of damage to internal thoracic organs, pelvic fracture, or one of the disorders of physiological parameters: GCS ≤ 8, RR < 10/min, or SBP ≤ 80 mmHg. In the other cases, the modification should also take into account the change in the GCS limit value to ≤ 14 (instead of ≤ 8) and the change in the SBP cutoff value from ≤ 80 mmHg to a higher one (but ≤ 129 mmHg) or the use of another factor. To complete the formula (undertriage according to ISS = 73.47%), additional factors, such as the mechanism of injury, must be included. It is necessary to develop research on the criteria for the qualification of geriatric patients to the TC, and the use of weight analysis of individual factors may contribute to the identification of properly balanced triage criteria.

## Background

Trends in the profile of trauma victims are changing. Owing to the increase in life expectancy, the proportion of older people is increasing at an exponential rate. Studies report that, in the last decade, the population of people aged over 65 years in the U.S. has grown approximately five times faster than the general population has [1]. Similarly, the number of older victims is increasing at an exponential rate. Not long ago, it was reported that by 2050, older adults would account for 40% of all hospital admissions for injuries, and this figure was already exceeded in 2013 [2]. These trends determine the need to adapt trauma systems in terms of, among other things, ensuring that older individuals have adequate accessibility to specialized units such as trauma centers (TCs).

However, geriatric age, which has been recognized as an independent risk factor for the death of injured patients, is challenging in terms of refining algorithms for the management of injured patients. This manifests itself in a high rate of undertriage, defining the underestimation of injuries among the oldest victims and the qualification of severe injury to lower referral centers [3]. The problem stems from specific pathophysiological processes that progress with age, resulting in a worse response of the body to injury, contributing to increased mortality or serious complications, especially in patients not included in the trauma system [4]. These peculiarities contribute to significant differences in scientific findings, making it difficult to develop an optimal triage protocol for geriatric patients [1]. This demonstrates the need for further development of research to find consensus in this important area, which also motivated the analysis of Polish standards of management.

The qualification for a TC in Poland is based on the provisions of the Regulation of the Minister of Health of June 18, 2010, which defines the criteria shown in Table 1 [5].

**Table 1:** The current TC qualification criteria

*Anatomical injuries*	
1	Penetrating wounds to the head or torso or blunt trauma with symptoms of damage to internal organs of the head, chest and abdomen
2	Amputation of an extremity above the knee or elbow
3	Extensive crush injury of extremities
4	Spinal cord injury
5	Fracture of an extremity with vascular and nerve damage
6	Fracture of two or more proximal long bones of the extremities or pelvis
*Physiological parameters disorders*	
1	Systolic blood pressure ≤ 80 mmHg
2	Pulse ≥ 120 per minute
3	Respiratory rate < 10 or > 29 per minute
4	Level of consciousness on the GCS scale ≤ 8
5	Arterial blood saturation ≤ 90%

To qualify a patient for TC, it is necessary to find at least 2 specific anatomical injuries and at least 2 abnormalities in physiological parameters, that is, to meet a minimum of 4 conditions. These criteria are applied to all adult trauma patients without considering the specificity of geriatric age [5].

The aim of this study was to verify the Trauma Center qualification criteria for patients over 65 years old (65 +) and analyze the weights of individual triage factors for these patients.

The research hypotheses assume that the current TC qualification criteria are not complete criteria for selecting 65+ patients and need to be modified to ensure precision in qualifying these patients.

## Materials and methods:

In this study, a retrospective analysis of the medical records of ED patients from the Clinical Hospital with Trauma Center, admitted from 1 Jan. 2017 to 31 Dec. 2020 was performed.

Study stages:

1. From a core group of 155,320 patients, comprising the total number of patients admitted to the ED, 6541 who were injured 65+, i.e., trauma group 65+, were selected on the basis of ICD-10 diagnoses, and a TRISS score for each of these patients was determined.
2. A group of 39 patients meeting the current TC qualification criteria, i.e., the TC 65+ group, was selected.
3. The TRISS cutoff value was determined, and on this basis, the TRISS 65+ group (94 patients) and the TC-omitted 65+ group (55 patients) were selected, indicating the degree of leakage of the TC qualification criteria.
4. The statistical significance and weighting of the current TC qualification factors were estimated, and the extent of modification of the values of relevant physiological parameters was determined.

Receiver operating characteristic (ROC curve) analysis was used for statistical classification; the TRISS score (percentage) was used as the independent variable. A 5% error of inference and an associated statistical significance level of *p* = 0.05 were assumed, indicating the presence of statistically significant differences or relationships. Multivariate linear regression analysis was used to statistically analyze the individual factors, which enabled the identification of relationships between the continuous independent variables, i.e., the TRISS scale score, and the dependent variables, which are the values of the individual classification factors. Owing to the lack of a clear definition of geriatric age, age 65 (65+) was adopted as the most commonly used age range in research studies [6].

The trauma and injury severity score (TRISS) is used to classify the clinical condition of patients and is the most effective tool for assessing the degree of trauma associated with 65+ injuries [7]. The TRISS scale predicts the survival rate of injured patients on the basis of a combination of anatomical and physiological trauma scales. The subscales used are the Injury Severity Score (ISS) and a coded version of the Revised Trauma Score (RTS). The TRISS differentiates between blunt and penetrating injury types and includes an age factor equal to 1 in patients ≥ 55 years of age and 0 < 55 years of age. The result of the TRISS scale is the probability of survival index (Ps), obtained as a percentage [3]. Injuries assessed according to the ISS scale are analyzed in relation to six body regions, defined as follows: head or neck, face, chest, abdominal or pelvic contents, extremities or pelvic girdle, and external structures.

To calculate the ISS score, the highest value of the abbreviated injury score (AIS) is selected in each of the three most injured areas of the body, after which each value is squared and summed: ISS = (1st AIS index)^2^ + (2nd AIS index)^2^ + (3rd AIS index)^2^. A description of injury severity according to the AIS index [8] is presented in Table 2.

**Table 2:** AIS injury severity indices

No	AIS scale	
	AIS index	Injury severity description
1.	1	Minor
2.	2	Moderate
3.	3	Serious
4.	4	Severe
5.	5	Critical
6.	6	Maximal
7.	9	Unknown

## Results

### Trauma group 65+

**Fig. 1:**
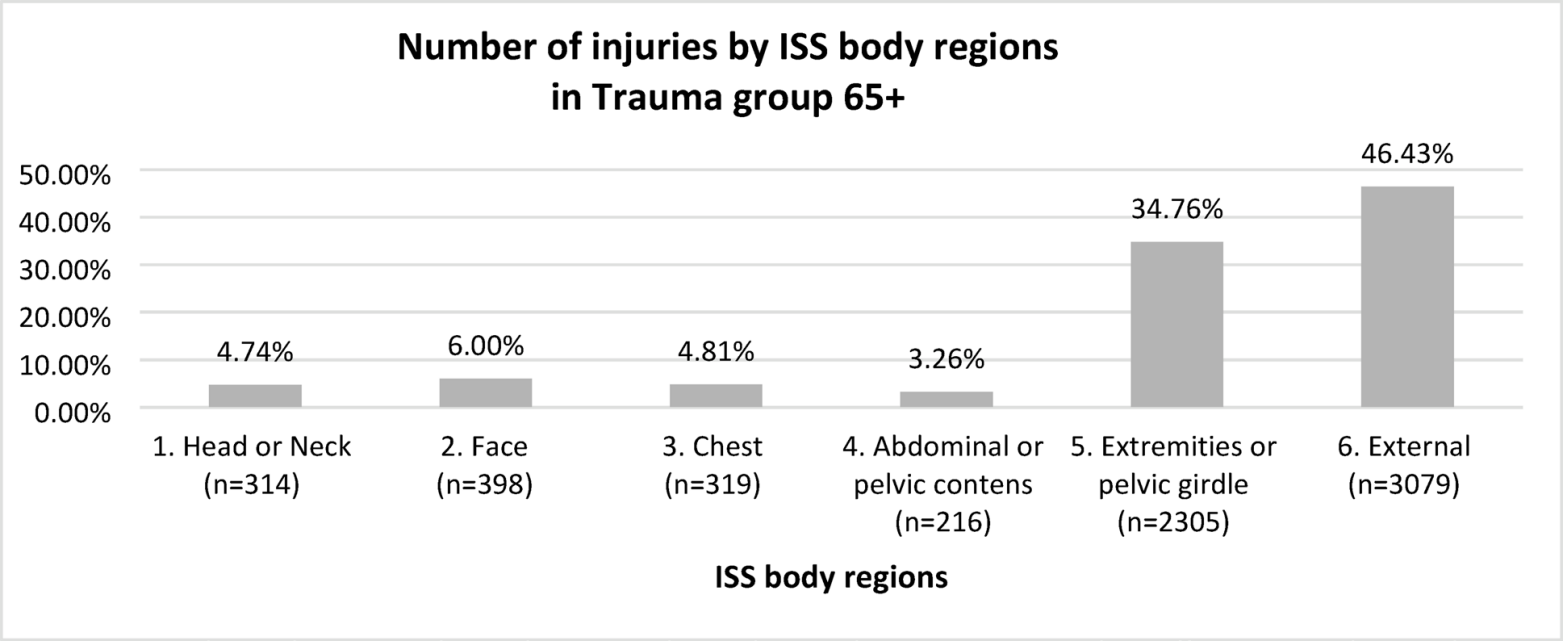
Injury characteristics by ISS regions, in trauma group 65+

From the core group of 155,320 patients aged 1–102 years (mean age: 49 years, SD: 21; number of women: 77,216, i.e., 49.71%, number of men: 78,102, i.e., 50.28%), on the basis of ICD-10 diagnoses, 6541 65+ patients admitted to the ED after trauma (trauma group 65 +) were selected. In the trauma group 65+, the mean age was 77 years (SD: 8.48), and the predominant gender was female (4199), accounting for 64.20%, with men (2342), accounting for 35.80% of the group. The injury characteristics of trauma group 65+ patients according to ISS regions, are shown in Fig. 1.

In the trauma group 65+, injuries in 2 regions predominated by far, i.e., external and extremities or pelvic girdle, accounting for 81.19% of the injuries reported in this group.

**Fig. 2:**
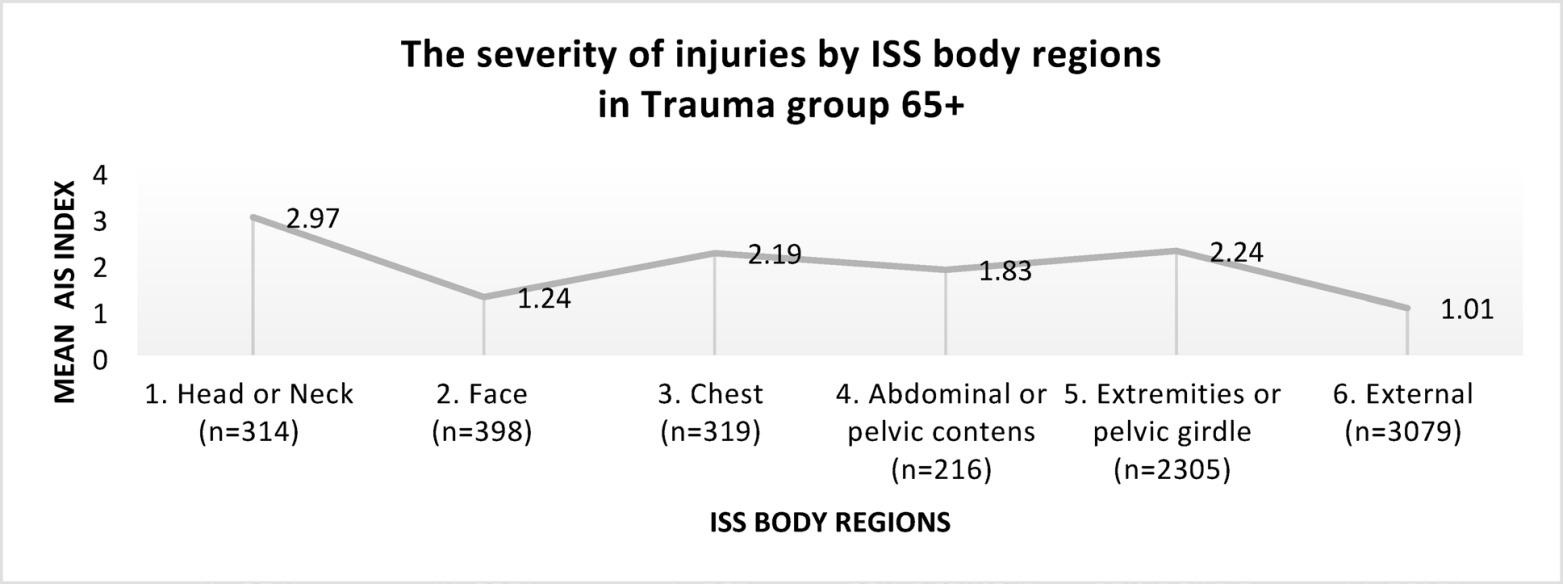
The severity of injuries by ISS regions, in trauma group 65+

The severity of injuries in trauma group 65+ according to ISS regions is shown in Fig. 2.

In the trauma group 65+, the most severe injuries, according to the mean AIS index, occurred in the following regions: head or neck, extremities or pelvic girdle, and chest.

The basic statistics of the TRISS scores in the trauma group 65+ are shown in Table 3.

**Table 3:** TRISS score statistics for the study groups

Group	Mean TRISS (SD)	Minimum TRISS	Maximum TRISS
Trauma group 65+	97.17% (SD: 0.07)	0.42%	98.31%
TC 65+ group	34.58% (SD: 0.32)	0.42%	88.84%
TRISS 65+ group	49.98% (SD: 31.26)	0.42%	88.84%
TC-omitted 65+ group	59.19% (SD: 26.49)	7.00%	88.68%

### TC 65+ group

**Fig. 3:**
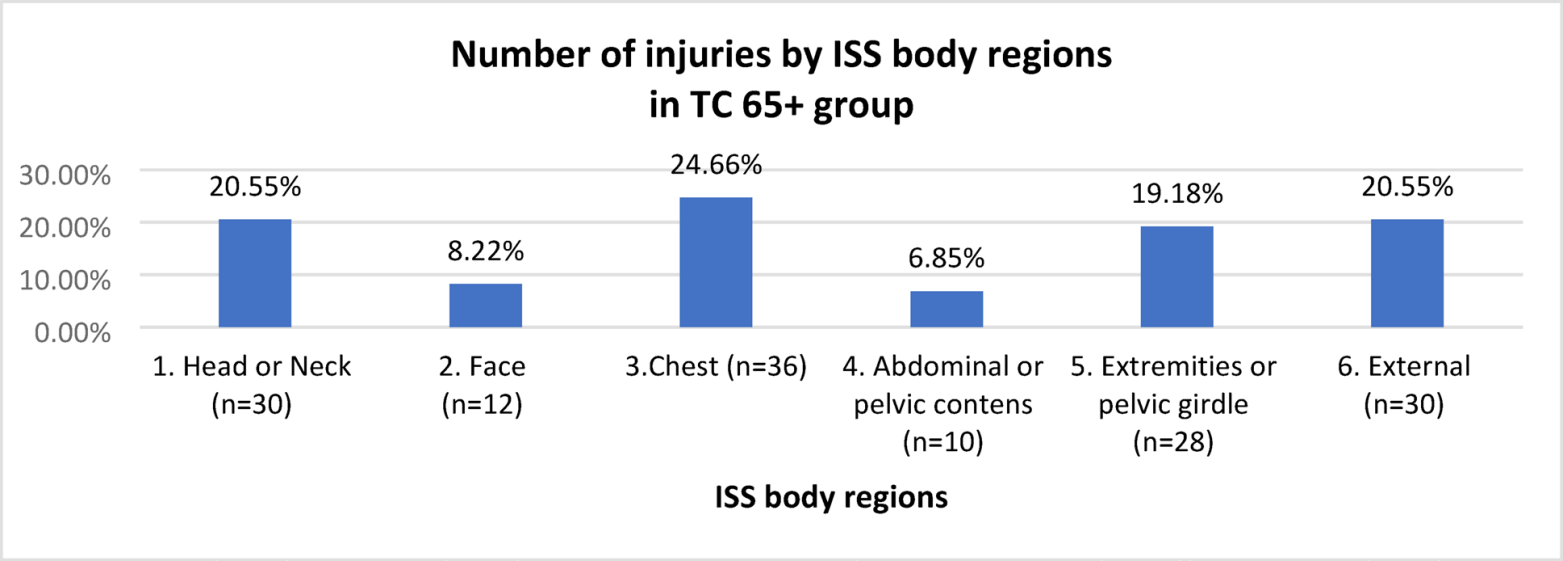
Injury characteristics by ISS regions, in the TC 65+ group

Thirty-nine patients were selected from the trauma group 65+ and met the current TC qualification criteria (TC 65+ group). In the TC 65+ group, the mean age was 74 years (SD: 7.13), and the predominant sex was male (n = 27; 69.23%), with 12 (30.77%) females. The injury characteristics of the TC 65+ group, according to ISS regions, are shown in Fig. 3.

In the TC 65+ group, the predominant injuries were in the following regions: chest, head or neck, and external, accounting for 64.39% of the injuries reported in this group.

**Fig. 4:**
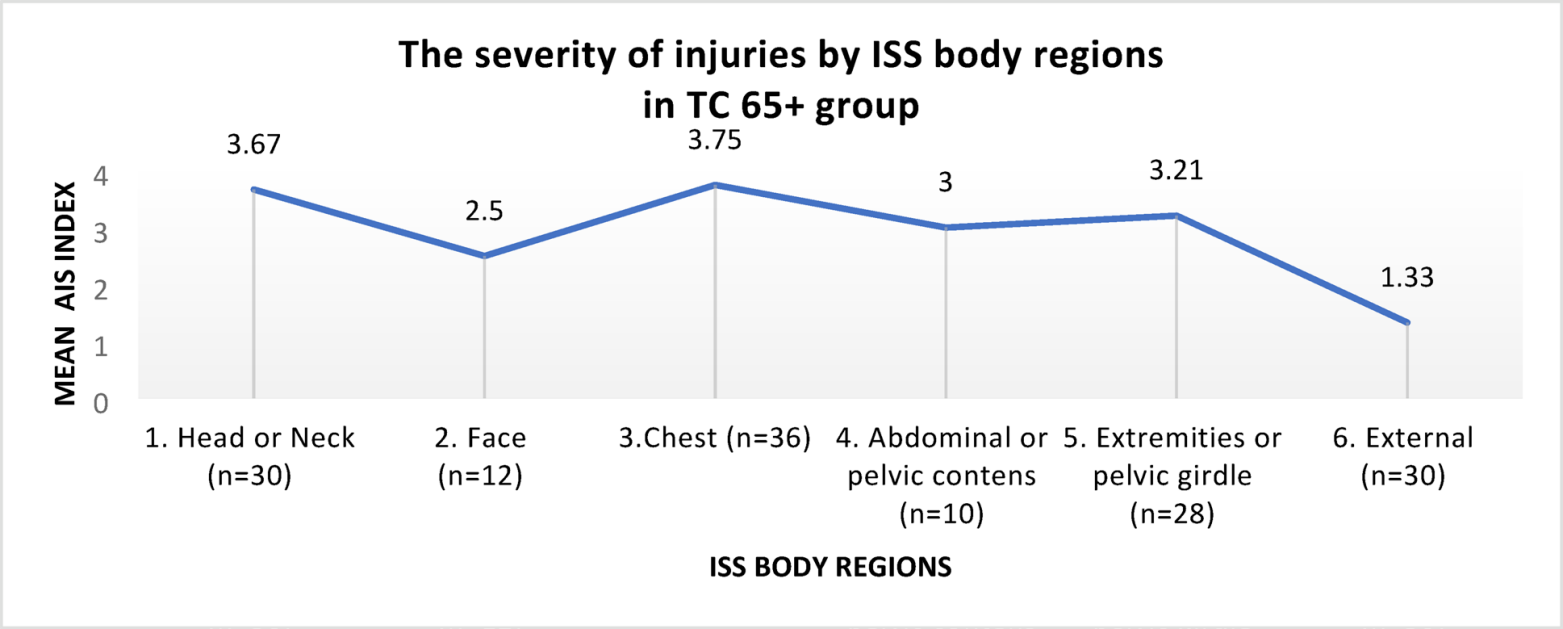
The severity of injuries by ISS regions, in the TC 65+ group

The severity of injuries in the TC 65+ group, according to ISS regions, is shown in Fig. 4.

In the TC 65+ group, the most severe injuries, according to the mean AIS index, occurred in the following regions: chest, head or neck, extremities or pelvic girdle.

The basic statistics of the TRISS scale scores in the TC 65+ group are shown in Table 3.

### TRISS 65+ group (TRISS cutoff point)

On the basis of the ROC analysis, the TRISS cutoff point for qualifying patients for TC was selected in the trauma group 65+. The results of the ROC analysis are shown in Table 4.

**Table 4:** Results of the ROC analysis in the trauma group 65+

Scale	Cutoff	Sensitivity	Specificity	PPV	NPV	AUC	Youden Index	*p* value
TRISS	0.89	1.000	0.991	39	6441	0.997	0.991	0.0000

The obtained TRISS cutoff point of 88.84% indicates that a score lower than or equal to this value should qualify a patient for TC. On the basis of the TRISS cutoff value obtained, 94 injured patients were selected out of 6541 patients, who should be referred to the TC (TRISS 65+ group). In the TRISS 65+ group, the mean age was 76 years (SD: 8.00), and the predominant sex was male (n = 60; 63.83%), with 34 (36.17%) female patients.

The basic statistics of the TRISS scale scores in the TRISS 65+ group are shown in Table 3.

### TC-omitted 65+ group (undertriage index based on TRISS)

**Fig. 5:**
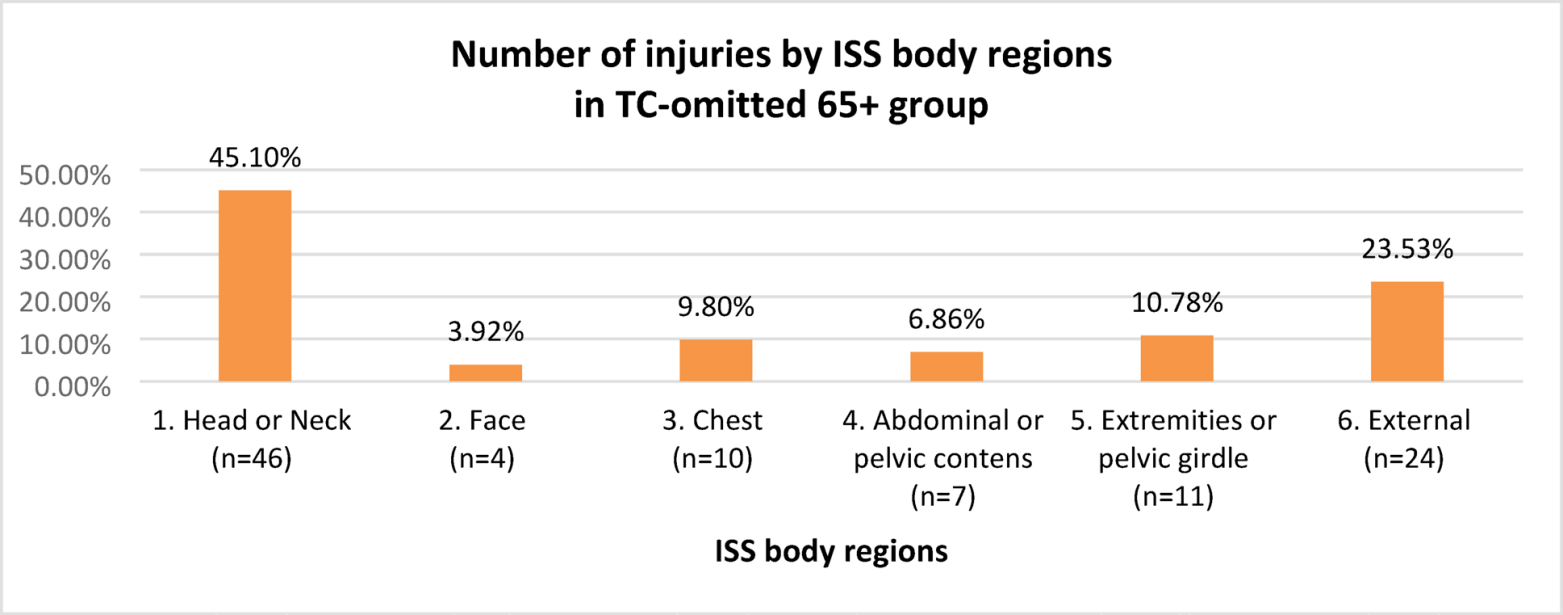
Injury characteristics by ISS regions, in the TC-omitted 65+ group

A comparison of the TRISS 65+ group with the TC 65+ group yielded a difference of 55 patients, accounting for 58.51% of the cases. These results confirmed the incompleteness of the current TC qualification criteria, i.e., the existence of a group of patients who should have been placed in the TC 65+ group, but owing to the “leakiness” of the criteria, they were not qualified for it, forming a group of injured persons omitted from the TC qualification (the TC-omitted 65+ group). In the TC-omitted 65+ group, the mean age was 77 years (SD: 8.31), and the predominant sex was male (n = 33; 60%), with female sex (n = 22; 40%). The injury characteristics of the TC-omitted 65+ group according to ISS regions, are shown in Fig. 5.

In the TC-omitted 65+ group, the predominant injuries were in the following regions: head or neck, external, and extremities or pelvic girdle, accounting for 79.41% of the injuries reported in this group.

**Fig. 6:**
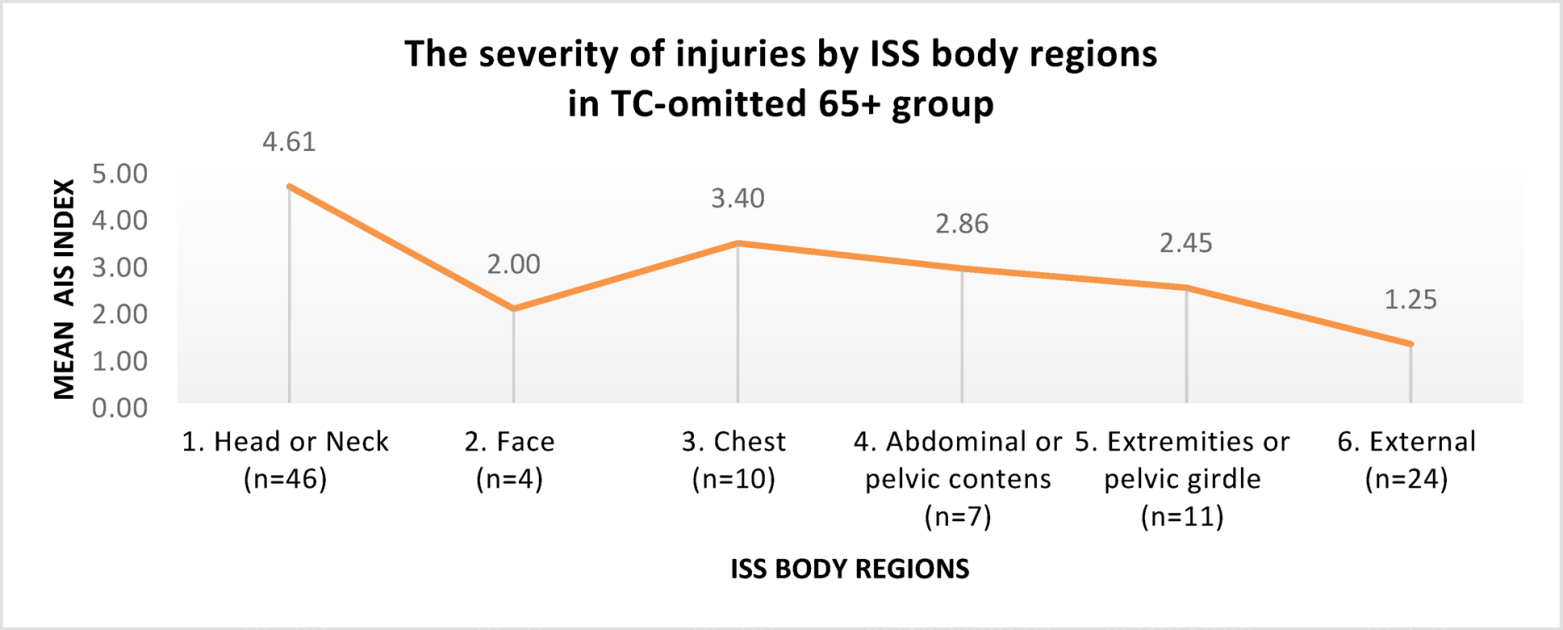
The severity of injuries by ISS regions, in the TC-omitted 65+ group

The severity of injuries in the TC-omitted 65+ group, according to ISS regions, is shown in Fig. 6.

In the TC-omitted 65+ group, the most severe injuries, according to the mean AIS index, occurred in the following regions: head or neck, chest, and abdominal or pelvic contents.

The basic statistics of the TRISS scores in the TC-omitted 65+ group are shown in Table 3.

**Fig. 7:**
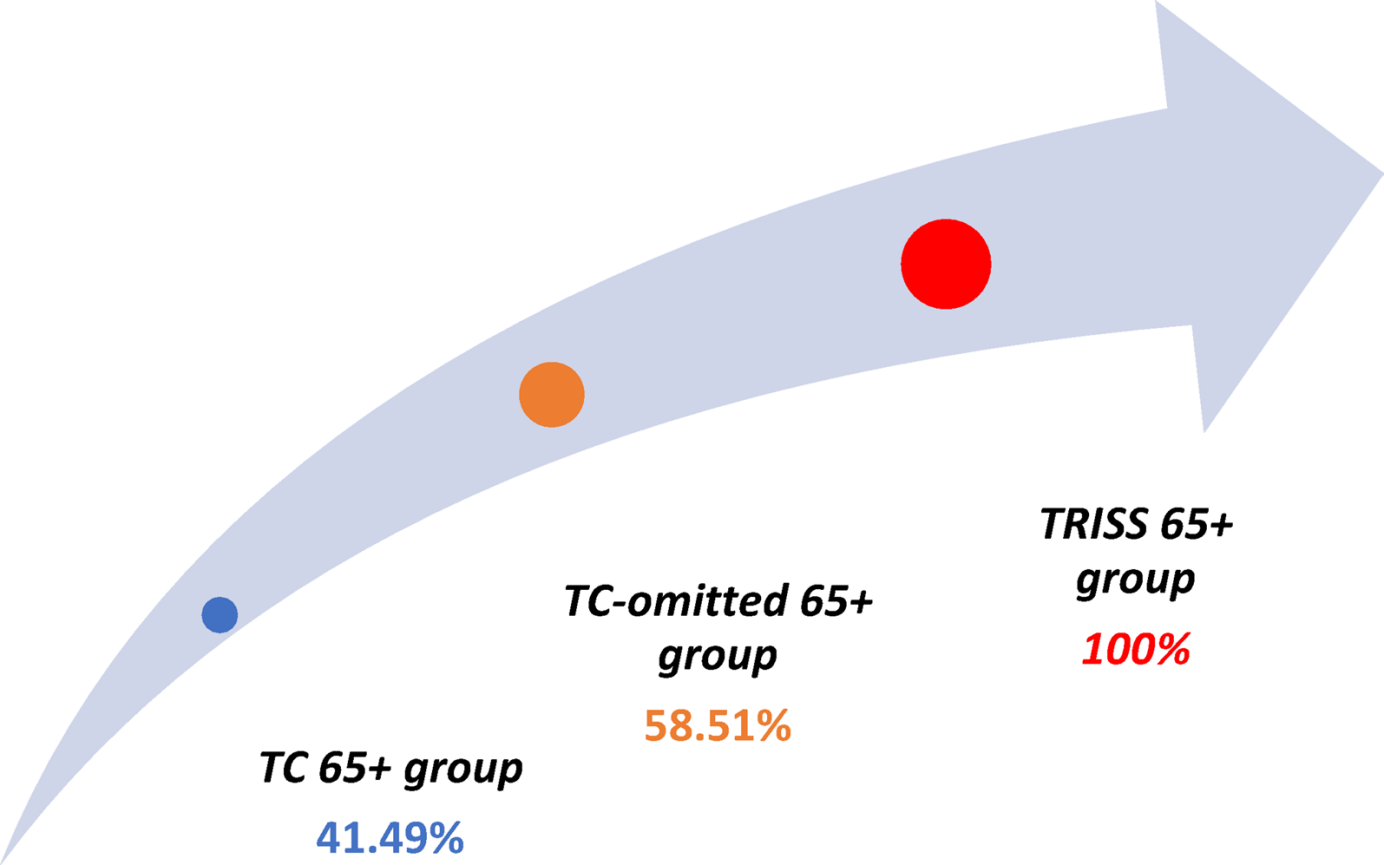
Correlations between the 65+ groups

The relationships between the TC-omitted 65+ group and the TRISS 65+ and TC 65+ groups are shown in Fig. 7.

The results obtained determine the degree of leakage of the current TC qualification criteria, which is also the undertriage rate estimated according to TRISS at 58.51%.

**Fig. 8:**
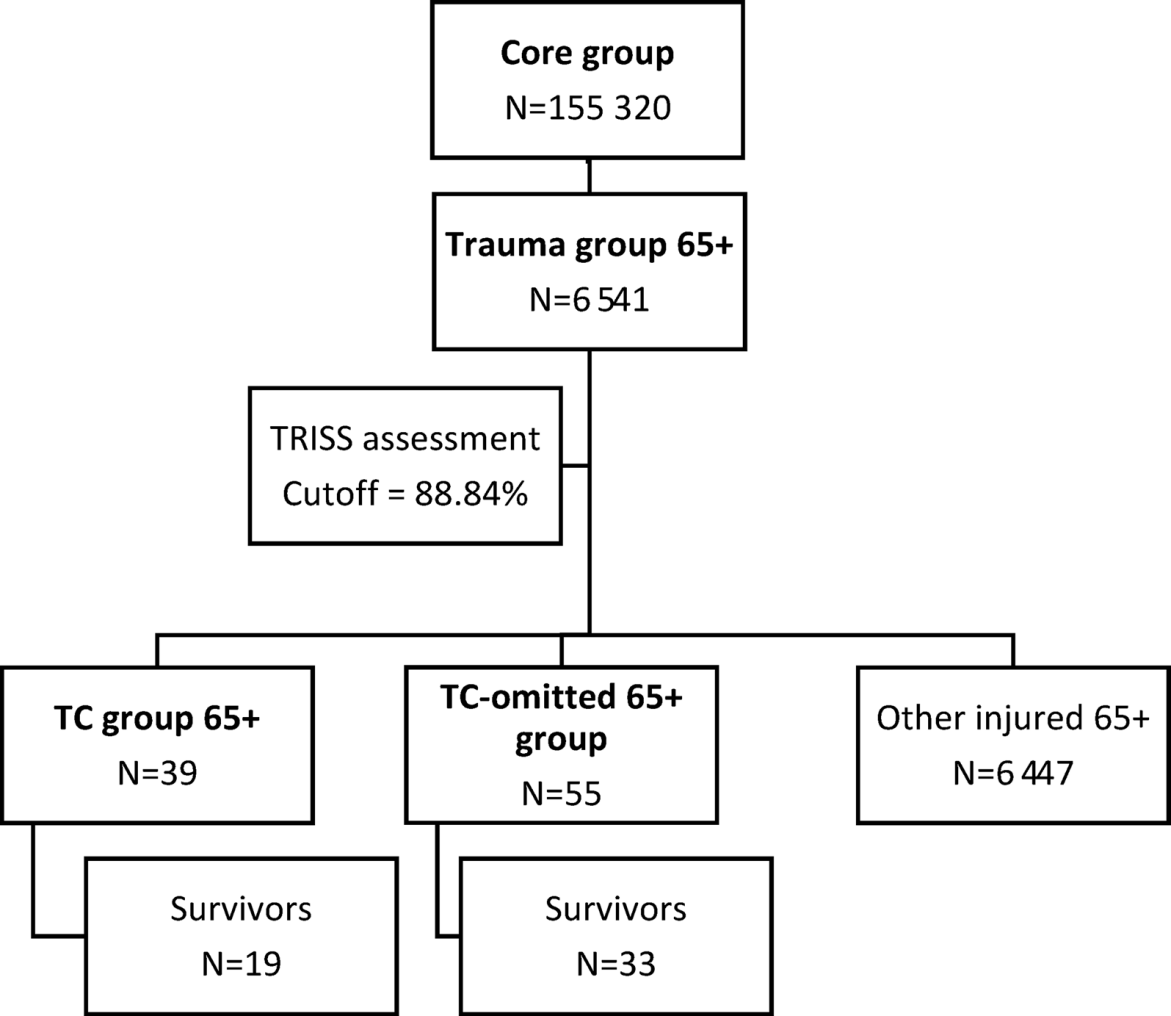
Flow chart of the patient groups included in the study

The summary characteristics of the patient groups included in the study are shown in Fig. 8.

### Modification of the TC qualification criteria

As a result of the multivariate linear regression analysis, the statistical significance of the TC qualification criteria was estimated. Among the 11 items specified in the current TC qualification criteria (Table 1), 16 factors were extracted and analyzed separately, i.e., each injury and physiological parameter was treated separately, without giving it additional weights. Owing to the lack of occurrence of two types of injuries, i.e., penetrating wounds to the trunk and amputation of a limb above the elbow, these factors were excluded from the statistical analysis. A statistically significant *p* value was determined for each factor. All factors with *p* values < 0.05 were considered statistically significant (Table 5).

**Table 5:** Results of regression analysis of TC qualification factors

No	TC qualification factors	*p* value
1.	Penetrating head wounds	0.061808
2.	Blunt injuries with symptoms of damage to internal organs of head	0.123637
3.	Blunt injuries with symptoms of damage to internal organs of thorax	**0.000473**
4.	Blunt injuries with symptoms of damage to internal organs of abdomen	0.670304
5.	Amputation of an extremity above the knee	0.113201
6.	Extensive crush injury of extremities	**0.010017**
7.	Spinal cord injury	0.277737
8.	Fracture of an extremity with damage to vessels and nerves	0.946686
9.	Fracture of at least two proximal long bones of extremities	0.555911
10.	Pelvic fracture	**0.010709**
11.	Systolic blood pressure ≤ 80 mmHg	**0.012204**
12.	Heart rate ≥ 120/min	0.140904
13.	Respiratory rate < 10/min	**0.000913**
14.	Respiratory rate > 29/min	0.209531
15.	Level of consciousness on the GCS scale ≤ 8	**0.000000**
16.	Arterial blood saturation ≤ 90%	0.950000

Bold values are used to highlights the results that are statistically significant or otherwise considered key findings in the analysis

As a result of the analysis, 6 factors that are statistically significant in the selection of patients aged 65+ to TC were identified. The result of the regression analysis is a list of regression coefficients (“b”) established for each factor of qualification for TC, determining the value by which the TRISS result decreases, i.e., the probability of survival of the patient, at the occurrence of a given factor. This action allows both the estimation of which factors have the strongest impact on the TRISS result at the statistical level and the determination of the estimated TRISS result (TRISSe). The weight of the impact of each factor on the qualification of patients 65+ to TC on the basis of the TRISS results is presented in Table 6. The factors are ordered according to the increasing effectiveness of patient typing.

**Table 6:** Weight of individual factors in qualification for TC

No	TC qualification factors	Regression coefficient "b” (%)	SD
1.	Systolic blood pressure (SBP) ≤ 80 mmHg	− **10.41%**	0.040566
2.	Pelvic fracture	− **11.35%**	0.043378
3.	Blunt injuries with symptoms of damage to internal organs of thorax	− **13.75%**	0.037616
4.	Respiratory rate (RR) < 10/min	− **16.16%**	0.046837
5.	Extensive crush injury of extremities	− **33.74%**	0.127763
6.	Level of consciousness on the GCS scale ≤ 8	− **42.90%**	0.041096

Bold values are used to highlights the results that are statistically significant or otherwise considered key findings in the analysis

**Fig. 9:**
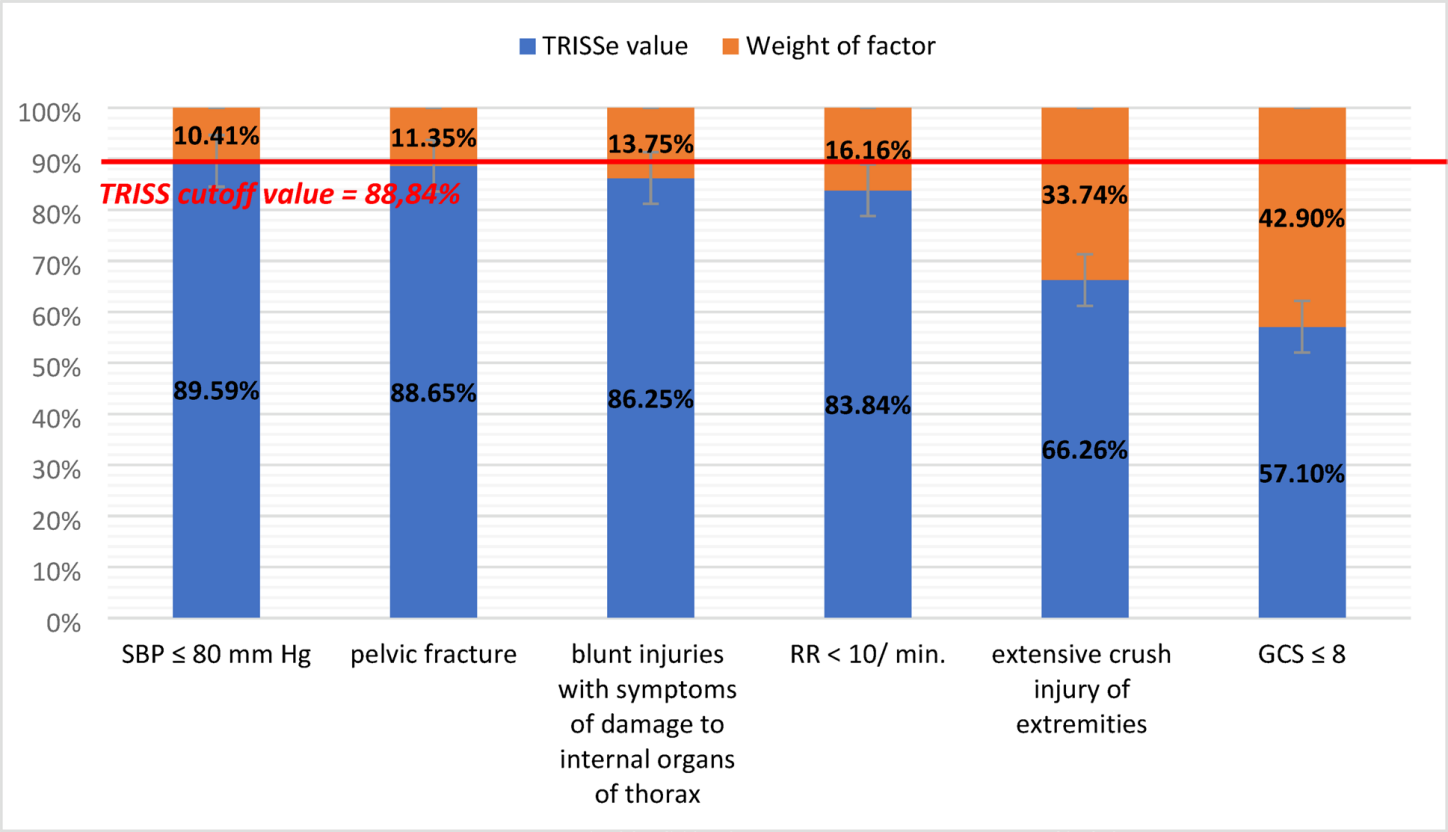
Weight of factors with respect to the TRISS cutoff value

The weights of the factors with respect to the TRISS cutoff value are shown in Fig. 9.

Differences in the weights of individual factors when qualifying for TC are significant. The weight of GCS ≤ 8 is greater than the sum of the weights of other physiological factors, including more than 2.5 times the weight of RR < 10/min. and more than 4 times the weight of SBP ≤ 80 mmHg. This result may be a premise for the modification (weakening) of selected factors to “balance” the TC qualification criteria for 65+ victims. Moreover, the weight and standard deviation (SD) of every statistically significant factor potentially affect the reduction in the TRISS_e_ value below the assumed cutoff value, i.e., 88.84% (Fig. 9), which can be the basis for reducing the number of required factors when qualifying for a TC.

In addition to the individual influence of factors on the value of TRISS_e_, their weights can be summed. The mutual amplification of factors and their impact on the final value of TRISS_e_ is expressed as the sum of all “b” values for factors occurring in a given patient. For example, the occurrence of a GCS score ≤ 8 (− 42.90%) and extensive crushing of extremities (− 33.74%) in a patient potentially reduces the TRISS_e_ score by 76.64% (TRISS_e_: 100% − 42.90% − 33.74% = 23.36%).

### Modification of the range of values of physiological parameters

Using ROC analysis, in the trauma group 65+, cutoff values for important physiological parameters, i.e., SBP, RR, and GCS, were determined in relation to TC qualification according to the value of the TRISS (TRISS 65+ group). The results of the analysis are presented in Table 7.

**Table 7:** ROC analysis results for the SBP, RR, and GCS factors

Factor	Cutoff	Sensitivity	Specificity	PPV	NPV	AUC	Youden Index	*p* value
SBP (mmHg)	129	0.574	0.840	54	5418	0.671	0.415	**0.0000**
RR (‘/min.)	15	0.415	0.994	39	6409	0.528	0.409	0.5606
GCS	14	0.798	0.982	75	6334	0.896	0.780	**0.0000**

Bold values are used to highlights the results that are statistically significant or otherwise considered key findings in the analysis

Among the outcomes of the analysis, the RR factor was not statistically significant (*p* value > 0.05).

The SBP result was statistically significant (*p* value < 0.05), but the cutoff value of 129 mmHg did not result in a satisfactory level of sensitivity (57.45%) or specificity (84.04%), with an AUC = 0.671. The poor predictive value of the obtained result is also evidenced by the low value of the Youden index = 0.415. Moreover, the result obtained is at the same time a typical value of a standard of dubious effectiveness as a differentiating criterion.

A GCS score of 14 had the best ratio of sensitivity (79.79%) and specificity (98.25%), with an AUC = 0.896. The high predictive value is also evidenced by the value of the Youden index = 0.780.

## Discussion

Clear signals about the need to improve the rules for the qualification of geriatric patients for TC in existing emergency medical systems have appeared in recent years, and most of the analyses devoted to this topic date from the past decade [9–16]. The rationale for addressing this issue in the context of the Polish guidelines may already be the low percentage of 65+ patients meeting the current TC qualification criteria (0.6% in relation to 6541 patients 65+ admitted to the ED for trauma) [5]. Some justification for limiting the number of patients admitted to the TC via the ED may be the struggle of these emergency units with the problem of overcrowding [17] or concerns about overtriage, i.e., being overwhelmed with patients without severe trauma [1]. However, regardless of the organizational assumptions, the rules for qualifying geriatric patients cannot be selective and must allow for consistent selection of patients with a certain severity of clinical condition. In contrast, the results of the present study show the inaccuracy of the Polish criteria in qualifying older victims for TC. This is evidenced by the omission of 58.51% of patients who, owing to the severity of their injury (according to the TRISS scale), should have been placed in the TC but were denied this opportunity owing to their failure to meet the conditions set forth in the current TC qualification criteria [5]. The results obtained determine the degree of leakage in the formula of the current TC qualification criteria, which is also the undertriage rate estimated according to the TRISS scale.

In addition, the overall undertriage rate in trauma group 65+ estimated according to another commonly used method, i.e., the percentage of ISS scores > 15 among patients disqualified from TC [1], would be 73.47%.

### Undertriage

The vast majority of recent studies on the medical segregation of older victims clearly indicate undertriage, i.e., underestimation of this group of patients when qualifying for TC [6, 12, 15, 18–24]. Published studies show variation in the degree of undertriage, which reaches the highest values in the oldest victims [15, 24–26], up to 62% over the age of 90 [15]. These findings are alarming, especially compared with the American College of Surgeons Committee on Trauma (ACS-COT)-recommended undertriage rate of < 5% in older individuals.

### Probability of survival

The results confirm that Poland's qualification criteria for TC are too restrictive and consider only victims with the most severe injuries and minimal chances of survival [27]. Taking into account the mean probability of survival according to the TRISS in the TC 65+ group, which was 34.58% (SD: 0.32), one can conclude that in the current reality, older patients with severe injuries of the body have a poor chance of survival regardless of whether they meet the qualification criteria for TC or are disqualified by them. Moreover, the higher mean TRISS value in the TRISS 65+ group (49.98%, SD: 31.26) indicates that modifying the current TC qualification criteria may reduce undertriage and improve the survival rate of injured 65+ individuals.

In published studies, undertriage is cited as a major cause of decreased survival probability for older trauma patients [15, 28]. Among geriatric victims with undertriaged injuries, higher rates of mortality (21% vs. 6%), disability (22% vs. 6%) and complications (39% vs. 23%) are reported than among younger adult victims [15]. Despite the increase in posttraumatic mortality among older individuals with increasing age [29], fewer deaths are recorded in the TC than in other hospital units (20% vs. 32%) [15, 18]. While published results are not entirely consistent, they indicate that, given appropriate comorbid conditions specific to older individuals, referral to TC can be associated with significantly lower (up to 41%) 30-day mortality rates for 65+ patients [21, 30], as well as lower annual mortality rates [1, 15, 31]. However, evidence-based, precise algorithms for qualifying geriatric casualties for TC remain a challenge, and the mechanisms for segregating these patients remain controversial [1]; therefore, research aimed at reducing trauma mortality is focused on developing an effective method for segregating casualties for TC [6, 15, 21, 26, 28, 29].

### Factors of TC qualification criteria for geriatric patients

Research by various research centers is currently focused on finding optimal criteria for segregating geriatric patients, either by adding appropriate modifications to existing guidelines or developing separate procedures, which are based mostly on the Field Triage Scheme and the Ohio Adult Triage Criteria. These works are the source of at least 8 concepts for prehospital triage systems specific to older victims [1, 9–12, 14–16, 23, 32].

The present study, which verifies the accuracy of the assumptions of the current TC qualification criteria [5], identified 6 factors relevant to the qualification of geriatric patients for TC (SBP ≤ 80 mmHg, pelvic fracture, blunt injuries with symptoms of damage to internal thoracic organs, RR < 10/min, extensive crush injury of extremities, GCS ≤ 8), but with different typing weights for patients.

The results obtained are in line with the main trends presented by other authors referring to this topic. All emerging triage systems dedicated to the geriatric population include specific physiological parameters [9, 10, 12, 14, 33–35], and the most commonly identified vital signs associated with serious injuries are GCS, SBP and RR [16]. In addition, most of these triage systems consider geriatric-specific anatomical injury factors, which often occur in conjunction with the mechanism of injury [9, 10, 12, 14, 33–35]. A lack of consideration of the mechanism of injury is an element that significantly differentiates Polish TC qualification criteria from other systems for segregating the injured [27].

## Physiological parameters

### SBP

In the present study, a cutoff value of an SBP of 129 mmHg was obtained, with questionable differential efficacy for qualifying patients for TC; nevertheless, one study suggested that an SBP < 130 mmHg may increase the risk of death in older patients [7]. In addition, all established systems for segregating geriatric victims have used an SBP criterion with cutoff values higher than 80 mmHg [14]. SBP < 90 mmHg in older victims was found to increase mortality by 3–5 times [1]. The values proposed in triage systems for older victims vary and are < 90 mmHg (in at least 2 systems), < 100 mmHg (in 1 system) and < 110 mmHg (in more than 5 systems) [1, 14, 16, 32–34, 36].

Decisions about introducing the described changes have been justified by the tendency in older patients for their condition’s apparent stability despite the lack of reflection in the actual physiological state [33]. The significant differences in SBP in the study groups have been linked to the “relative hypotension” typical in older individuals, which occurs despite apparently “normal” blood pressure values [6, 32, 37]. More than one-third of geriatric trauma patients with SBP > 90 mmHg have been found to have latent hypoperfusion, as determined by lactate levels or base deficits [26, 33]. One study revealed that the mortality rate of older victims with “normal” vital signs can reach 16% [6, 21]. These results support the recognition that the shock threshold in geriatric patients is lower than the typical threshold [1, 26].

The value of SBP < 100 mmHg was included, among other criteria, in one of the best studied and developed regional triage criteria, i.e., the 2019 Ohio Adult Triage Criteria, for patients ≥ 70 years of age [1, 9, 10, 12, 38].

In contrast, the current combined guidelines of the American College of Surgeons Committee on Trauma (ACS COT) and Centers for Disease Control and Prevention (CDC), which form the National Guidelines for the Field Triage of Injured Patients (Field Triage Scheme), updated in 2021, recommend transporting patients 65+ with SBP < 110 mmHg to a TC of the highest category [13]. This study revealed that the SBP threshold of < 110 mmHg when 65+ patients are classified improves sensitivity (13–29%) while maintaining specificity (83–93%) [13]. It has also been shown that using SB* p* values < 110 mmHg significantly reduces undertriage and only moderately increases overtriage [1, 15, 26, 33]. Moreover, these reports verified the suggestions of some authors about causing unacceptably high increases in the overtriage rate [16], which, according to ACS COT recommendations for older victims, should be in the range of 25–35% [6, 16].

#### Shock index

Given the failure to recognize hemorrhagic shock in older victims as the most common cause of preventable deaths [11, 26], some studies have proposed the inclusion of other nonstandard medical segregation factors, such as the shock index, which assesses the ratio of heart rate to SBP (HR/SBP) [26, 39, 40]. The introduction of this index was supported by results indicating that victims aged 65+ had lower heart rates and higher blood pressure than younger patients did, thus not meeting standard shock segregation criteria [1]. Notably, the shock index may be more helpful in predicting mortality in older individuals than separately used heart rate or SBP values are [6, 40], and this has also been taken into account in the Field Triage Scheme by including a shock index > 1 as a recommendation for top-level TC [1, 16].

### RR

In the present study, the results of the analysis determining the cutoff value of the RR factor were not statistically significant (*p* value > 0.05).

Some studies identifying relevant prehospital physiological factors for the segregation of older people with serious injuries recommend changing the standard respiratory rate range (< 10 or > 29/min). Two studies recommended a range of < 10 or > 24 breaths/min. as optimal limits for the respiratory rate in geriatric patients who are qualified for TC [14, 16].

Other researchers, including the authors of the Ohio Adult Triage Criteria [38] and the Field Triage Scheme, among others, recommend maintaining the typical respiratory rate range of < 10 or > 29/min, including for older patients. Notably, this range, in adult patients with serious injuries in general, achieves a sensitivity of 13% and specificity of 96%, with an AUC = 0.70, and shows good predictive utility in older patients as well [13].

### GCS

The results of the present study revealed that a GCS cutoff value of 14 had the best ratio of sensitivity (79.79%) and specificity (98.25%), with an AUC = 0.896.

Similarly, in other published studies, the assessment of the baseline GCS score was crucial for making decisions regarding the segregation of geriatric age victims [26, 36]. Recognizing that a low GCS score is associated with a poor prognosis and represents an independent risk of increased mortality, all emerging triage systems dedicated to older individuals have increased the GCS threshold for TC qualification [14]. This was justified by the possible initially higher GCS score in geriatric patients after trauma, which can rapidly deteriorate due to changes in the cerebral vasculature. This is related mainly to when the additional buffer capacity is filled by the growing intracranial hematoma [26, 41]. This prolonged deterioration of consciousness may favor disqualification of geriatric patients from immediate treatment in TC and may be responsible for the increased mortality associated with traumatic brain injury compared with patients in younger age groups [26, 42].

The GCS thresholds recommended in the studies were ≤ 13 or ≤ 14 [14], and four triage systems dedicated to older victims proposed changing the GCS cutoff from 13 to 14 [9, 12, 14, 16, 23, 34, 36]. The authors of this proposal showed that a GCS from 15 to 14 was associated with increased mortality, a higher risk of traumatic brain injury, and an increased incidence of endotracheal intubation [26, 36]. One study revealed that adopting a GCS threshold of ≤ 14 had a sensitivity of 59.2% and specificity of 85.1% [15, 16, 36]. In the Ohio Adult Triage Criteria, a GCS threshold of ≤ 14 for geriatric age was associated with the occurrence of TBI, and a GCS threshold of ≤ 13 was adopted for other injuries [38].

In contrast, some researchers remain skeptical about the reliability of scales for assessing consciousness in older individuals, alluding to common confounding factors associated with dementia or preexisting neurological disorders [26]. In the latest update of the Field Triage Scheme, the GCS factor (≤ 13) was completely replaced by “Inability to follow commands (motor GCS < 6) as an indication for top-level TC for all patients” [13].

Wasserman et al. focused on traumatic brain injury and recommended TC qualification for geriatric patients who fail to respond spontaneously or who do not comply with motor commands [14, 35].

Other authors have recommended the use of markers such as serum lactate levels and alkaline deficiency as alternative predictors of mortality [32].

### Anatomical injuries

In the present study, in the TC 65+ group, most injuries occurred in the chest and head or neck regions (45.21% of all injuries in this group), which, according to the mean AIS, were the most severe injuries in this group. In the TC-omitted 65+ group, injuries in the head or neck area predominated (45.10%), with the most severe injuries in this group on average. The head and chest were the two most frequently represented regions in geriatric patients after serious injuries, which was also reported in another study [43]. The noticeable prevalence of head injuries in the TC-omitted 65+ group may suggest the poor sensitivity of the TC qualification criteria in this area.

In addition, the present study identified pelvic fracture, blunt injuries with evidence of damage to the internal organs of the thorax and extensive crushing of extremities as important factors in the TC qualification criteria.

Among the emerging triage systems dedicated to geriatric patients, head injuries, fractures of long bones or pelvic bones [14] and thoracic injuries are the main anatomical injuries [27]. The greater exposure to severe thoracic and pelvic injuries in this group of patients than in younger victims is attributed mainly to traffic accidents. Older adults are also more likely to suffer severe chest injuries in low-speed accidents [26]. Compared with other adults with similar injury severity, thoracic injuries among patients aged 65+ are associated with a fourfold higher mortality rate [44], and some studies recommend considering rib fractures as serious injuries that increase mortality [26].

### Mechanism of injury

Given that anatomical and physiological criteria identify less than half of patients with serious injuries, the mechanism of injury has been recognized as both an important segregation factor [13] and an acknowledged predictor of serious injury [25, 27]. In most triage systems dedicated to older adults, an association of anatomical injury with the mechanism of injury has been applied, concerning, among other things, pelvic and limb fractures as a result of being hit by a motor vehicle or in an accident involving a motor vehicle [9–12, 14, 33]. Some further-reaching studies have shown an association of severe injuries in geriatric patients with passenger motor activity after a traffic accident, the impact angle of the vehicle and the number of injuries [45] and have recommended considering these factors in medical segregation criteria [26].

Similarly, the Ohio Adult Triage Criteria and Field Triage Scheme recommend considering factors such as the following:

- ejection (partial or total) from a vehicle,
- death of another passenger in the same vehicle,
- vehicle telemetry data consistent with serious injury, for all victims [13, 38].

According to an additional analysis conducted in the present study, pelvic fracture was caused by an accident involving a motor vehicle, mainly a pedestrian, in all patients in the TRISS 65+ group. The incomprehensible lack of consideration of the mechanism of injury as an important element in qualifying adult patients for TC has already been criticized by Polish researchers [27]. This situation is even more incomprehensible in relation to other Polish qualification criteria, i.e., for pediatric trauma centers (Regulation of the Minister of Health of 2016), which consider factors such as the following:

- death of another person in the same incident,
- falling out of a motor vehicle, being crushed or falling from a height of > 3 m,
- extrication time from a vehicle, collapse or debris is longer than 20 min.,

despite the absence of visible anatomical injuries and significant disturbances in physiological parameters assessed at the scene [46].

In addition, the Ohio Adult Triage Criteria use the association of falls from any height with TBI as an indication of TC among geriatric age victims [38], whereas in the Field Triage Scheme, a fall combined with a “significant blow to the head” is considered a moderate-risk criterion warranting TC, but not necessarily the highest category [13].

### Other clinical factors

In addition to the described factors for the medical segregation of geriatric patients in terms of physiological parameters, anatomical injury and the mechanism of injury, other elements of triage are also being analyzed. Factors such as patient comorbidities or the use of anticoagulants have been added to three of the eight triage systems dedicated to older victims [14]. Owing to the low specificity of the results obtained, despite the demonstrated decrease in undertriage [26], the use of anticoagulants has not been considered a strict predictor in the qualification of geriatric patients for TC [14, 16]. Some studies indicate that the use of newer generations of these drugs is safer than the use of older formulations in the context of injury [16]. The Field Triage Scheme and Ohio Adult Triage Criteria allow EMS discretion with respect to triaging patients taking anticoagulants [1, 38].

Some studies have also reviewed the recommendation of direct transport to the TC of all victims aged 65+ with comorbidities, as proposed by the Eastern Association for the Surgery of Trauma (EAST) [47]. While several publications have shown a reduction in mortality as a result of complying with these recommendations [20, 21], this has not warranted the recognition of comorbidities identified prior to trauma as an independent factor in the medical segregation of geriatric patients [26, 48].

### Weighting of TC qualification factors

In the present study, the highest weight among the physiological parameter disorders was given to the factor with a GCS ≤ 8, whose weight was greater than the sum of the weights of the other physiological factors. The highest weight among anatomical injuries was obtained by extensive crush injury to the extremities, whose weight was greater than the sum of the other anatomical factors. In addition, the weight + standard deviation (SD) of each statistically significant factor was shown to potentially reduce the TRISSe below the accepted cutoff value, i.e., 88.84% (Fig. 9), which may provide a rationale for reducing the requirement for 4 factors [5] and recognizing the independence of these factors in typing 65+ patients for TC.

The reduction in the number of qualifying factors for TC corresponds with solutions used in other triage schemes. According to the Field Triage Scheme, the qualification of a patient for TC is conditional on the presence of only one factor among both anatomical (except long bone fractures) and physiological criteria or the mechanism of injury [13, 25, 27]. Similar principles were adopted by developers of the Ohio Adult Triage Criteria, who further reduced the number of suspected bone fractures from two to one, tying this condition to trauma in the mechanism of a traffic accident [6, 9, 10, 12, 14, 15]*.*

### Limitations of the study

The first limitation of the present study is that it is a retrospective analysis of information contained in medical records. Another limitation of the study was the surprisingly low number of geriatric patients meeting the current TC qualification criteria. On the basis of a previous analysis of the subject literature taking into account many more study groups, such large disparities compared with national conditions were not expected. A particular limitation of the study was the difficulty associated with the manual calculation of the TRISS score and its components, which are not standardized at the time of patient admission to the ED and hence cannot be obtained directly from medical records. Reliable calculation of TRISS scores requires deep analysis of each clinical case description, which, with 6541 patients, is time-consuming.

## Conclusions

The current TC qualification criteria require modification for geriatric patients, which would complete the leakage, estimated at 58.51% according to the TRISS scale.

This leakage results, among others, from an underestimation of the weight of 6 triage factors to the TC, and the modification of the criteria should include a reduction in the current requirement of 4 factors to 1 and allow admission to the TC of a 65+ trauma patient with one of the following anatomical injuries:

- extensive crush injury of extremities,
- blunt injuries with symptoms of damage to internal thoracic organs,
- pelvic fracture

or one of the disorders of physiological parameters:

- level of consciousness on the GCS ≤ 8.
- RR < 10/min.
- SBP ≤ 80 mmHg.

In the other cases, the modification of the triage criteria to the TC for 65+ patients should also take into account the change in the GCS limit value to ≤ 14 (instead of the current ≤ 8).

Moreover, for 65+ trauma patients, changing the SBP factor cutoff value from ≤ 80 mmHg to a higher value (in the range of up to 129 mmHg) or the use of another factor, e.g., the shock index, is crucial; however, further research in this area is needed.

In addition, to match the TC qualification criteria formula to the standard of the best advanced trauma systems and further reduce the undertriage rate in older people (estimated at 73.47% according to ISS), it is important to include additional factors such as the mechanism of injury in the Polish TC qualification criteria.

It is necessary to develop research on the criteria for the qualification of geriatric patients to the TC, and the analysis of the weights of individual factors is a tool that allows for a direct connection of the TRISS scale value (estimated post factum) with the factors of real assessment at the scene. This method may contribute to finding properly balanced criteria, the use of which in clinical practice will reduce high undertriage of qualifications of geriatric patients to TC.

## Data Availability

No datasets were generated or analysed during the current study.

## Abbreviations

TC: Trauma center
AIS: Abbreviated Injury Scale
ED: Emergency department
RR: Respiratory rate
SBP: Systolic blood pressure
GCS: Glasgow Coma Scale
Ps: Probability of survival
PPV: Positive predictive value
NPV: Negative predictive value
AUC: Area under the curve
HR: Heart rate
ACS COT: American College of Surgeons Committee on Trauma
TBI: Traumatic brain injury
EMS: Emergency medical services
SD: Standard deviation

## References

[1] Egodage T, Ho VP, Bongiovanni T, Knight-Davis J, Adams SD, Digiacomo J, et al. Geriatric trauma triage: optimizing systems for older adults-a publication of the American Association for the Surgery of Trauma Geriatric Trauma Committee. Trauma Surg Acute Care Open. 2024. 10.1136/tsaco-2024-001395. 39021732 10.1136/tsaco-2024-001395 PMC11253746

[2] Jiang L, Zheng Z, Zhang M. The incidence of geriatric trauma is increasing and comparison of different scoring tools for the prediction of in-hospital mortality in geriatric trauma patients. World J Emerg Surg. 2020;15(1):59. 33076958 10.1186/s13017-020-00340-1 PMC7574576

[3] Madni TD, Ekeh AP, Brakenridge SC, Brasel KJ, Joseph B, Inaba K, et al. A comparison of prognosis calculators for geriatric trauma: a prognostic assessment of life and limitations after trauma in the elderly consortium study. J Trauma Acute Care Surg. 2017;83(1):90–6. 28422904 10.1097/TA.0000000000001506

[4] Wiles LL, Day MD. Delta alert: expanding gerotrauma criteria to improve patient outcomes: a 2-year study. J Trauma Nurs. 2018;25(3):159–64. 29742626 10.1097/JTN.0000000000000371

[5] Rozporządzenie. Ministra Zdrowia z 18 czerwca 2010 r. *w sprawie centrum urazowego* (Dz. U. nr 118, poz. 803).

[6] Warriner Z, Bernard AC. Geriatric trauma: triage guidelines. Curr Trauma Rep. 2020;6:125–32.

[7] Liu X-Y, Qin Y-M, Tian S-F, Zhou J-H, Wu Q, Gao W, et al. Performance of trauma scoring systems in predicting mortality in geriatric trauma patients: comparison of the ISS, TRISS, and GTOS based on a systemic review and meta-analysis. Eur J Trauma Emergency Surg. 2024:1–13. 10.1007/s00068-024-02467-1 38363328

[8] The Abbreviated Injury Scale © 2015 Revision. Chicago USA, 2016.

[9] Werman HA, Erskine T, Caterino J, Riebe JF, Valasek T, Board TCotSoOE. Development of statewide geriatric patients trauma triage criteria. Prehosp Disaster Med. 2011;26(3):170–9. 22107767 10.1017/S1049023X11006315

[10] Caterino JM, Brown NV, Hamilton MW, Ichwan B, Khaliqdina S, Evans DC, et al. Effect of geriatric-specific trauma triage criteria on outcomes in injured older adults: a statewide retrospective cohort study. J Am Geriatr Soc. 2016;64(10):1944–51. 27696350 10.1111/jgs.14376 PMC5117430

[11] Newgard CD, Richardson D, Holmes JF, Rea TD, Hsia RY, Mann NC, et al. Physiologic field triage criteria for identifying seriously injured older adults. Prehosp Emerg Care. 2014;18(4):461–70. 24933614 10.3109/10903127.2014.912707 PMC4397211

[12] Ichwan B, Darbha S, Shah MN, Thompson L, Evans DC, Boulger CT, et al. Geriatric-specific triage criteria are more sensitive than standard adult criteria in identifying need for trauma center care in injured older adults. Ann Emerg Med. 2015;65(1):92-100. e3. 24908590 10.1016/j.annemergmed.2014.04.019

[13] Newgard CD, Fischer PE, Gestring M, Michaels HN, Jurkovich GJ, Lerner EB, et al. National guideline for the field triage of injured patients: recommendations of the National Expert Panel on Field Triage, 2021. J Trauma Acute Care Surg. 2022;93(2):e49. 35475939 10.1097/TA.0000000000003627 PMC9323557

[14] Boulton AJ, Peel D, Rahman U, Cole E. Evaluation of elderly specific pre-hospital trauma triage criteria: a systematic review. Scand J Trauma Resuscitation Emerg Med. 2021;29(1):1–12. 10.1186/s13049-021-00940-z PMC8404299 34461976

[15] Alshibani A, Alharbi M, Conroy S. Under-triage of older trauma patients in prehospital care: a systematic review. Eur Geriatric Med. 2021;12(5):903–19. 10.1007/s41999-021-00512-5 PMC8463357 34110604

[16] Pandor A, Fuller G, Essat M, Sabir L, Holt C, Buckley Woods H, et al. Individual risk factors predictive of major trauma in pre-hospital injured older patients: a systematic review. Br Paramedic J. 2022;6(4):26–40. 10.29045/14784726.2022.03.6.4.26 PMC8892449 35340581

[17] Crawford K, Morphet J, Jones T, Innes K, Griffiths D, Williams A. Initiatives to reduce overcrowding and access block in Australian emergency departments: a literature review. Collegian. 2014;21(4):359–66. 25632734 10.1016/j.colegn.2013.09.005

[18] Cox S, Morrison C, Cameron P, Smith K. Advancing age and trauma: triage destination compliance and mortality in Victoria, Australia. Injury. 2014;45(9):1312–9. 24630836 10.1016/j.injury.2014.02.028

[19] Chang DC, Bass RR, Cornwell EE, MacKenzie EJ. Undertriage of elderly trauma patients to state-designated trauma centers. Arch Surg. 2008;143(8):776–81. 18711038 10.1001/archsurg.143.8.776

[20] Garwe T, Stewart K, Stoner J, Newgard CD, Scott M, Zhang Y, et al. Out-of-hospital and inter-hospital under-triage to designated tertiary trauma centers among injured older adults: a 10-year statewide geospatial-adjusted analysis. Prehosp Emerg Care. 2017;21(6):734–43. 28661712 10.1080/10903127.2017.1332123 PMC5668189

[21] Garwe T, Stewart KE, Newgard CD, Stoner JA, Sacra JC, Cody P, et al. Survival benefit of treatment at or transfer to a tertiary trauma center among injured older adults. Prehosp Emerg Care. 2020;24(2):245–56. 31211622 10.1080/10903127.2019.1632997 PMC6962564

[22] Nakamura Y, Daya M, Bulger EM, Schreiber M, Mackersie R, Hsia RY, et al. Evaluating age in the field triage of injured persons. Ann Emerg Med. 2012;60(3):335–45. 22633339 10.1016/j.annemergmed.2012.04.006 PMC3428427

[23] Newgard CD, Holmes JF, Haukoos JS, Bulger EM, Staudenmayer K, Wittwer L, et al. Improving early identification of the high-risk elderly trauma patient by emergency medical services. Injury. 2016;47(1):19–25. 26477345 10.1016/j.injury.2015.09.010 PMC4698024

[24] Horst MA, Morgan ME, Vernon TM, Bradburn EH, Cook AD, Shtayyeh T, et al. The geriatric trauma patient: a neglected individual in a mature trauma system. J Trauma Acute Care Surg. 2020;89(1):192–8. 32118822 10.1097/TA.0000000000002646

[25] Sasser SM, Hunt RC, Faul M, Sugerman D, Pearson WS, Dulski T, et al. Guidelines for field triage of injured patients: recommendations of the National Expert Panel on Field Triage, 2011. Morb Mortal Wkly Rep. 2012;61(1):1–20. 22237112

[26] Eichinger M, Robb HDP, Scurr C, Tucker H, Heschl S, Peck G. Challenges in the prehospital emergency management of geriatric trauma patients–a scoping review. Scand J Trauma Resuscitation Emerg Med. 2021;29:1–12. 10.1186/s13049-021-00922-1 PMC8305876 34301281

[27] Nowakowski J, Nowakowski R, Bilinski P, Nowak B, Wojciechowski P, Dworzynski M, et al. Comparison of American guidelines for field triage and Polish criteria as qualification to a trauma center. Ann Agric Environ Med. 2019. 10.26444/aaem/100538. 31559807 10.26444/aaem/100538

[28] Brown E, Tohira H, Bailey P, Fatovich D, Pereira G, Finn J. Older age is associated with a reduced likelihood of ambulance transport to a trauma centre after major trauma in Perth. Emerg Med Australas. 2019;31(5):763–71. 30827060 10.1111/1742-6723.13244

[29] Pracht EE, Langland-Orban B, Flint L. Survival advantage for elderly trauma patients treated in a designated trauma center. J Trauma Injury Infect Crit Care. 2011;71(1):69–77. 10.1097/TA.0b013e31820e82b7 21818016

[30] Candefjord S, Asker L, Caragounis E-C. Mortality of trauma patients treated at trauma centers compared to non-trauma centers in Sweden: a retrospective study. Eur J Trauma Emerg Surg. 2022;48(1):525–36. 32719897 10.1007/s00068-020-01446-6 PMC8825402

[31] MacKenzie EJ, Weir S, Rivara FP, Jurkovich GJ, Nathens AB, Wang W, et al. The value of trauma center care. J Trauma Injury Infect Crit Care. 2010;69(1):1–10. 10.1097/TA.0b013e3181e03a21 20622572

[32] De Simone B, Chouillard E, Podda M, Pararas N, de Carvalho Duarte G, Fugazzola P, et al. The 2023 WSES guidelines on the management of trauma in elderly and frail patients. World J Emerg Surg. 2024;19(1). 10.1186/s13017-024-00537-8 PMC11140935 38816766

[33] Brown JB, Gestring ML, Forsythe RM, Stassen NA, Billiar TR, Peitzman AB, et al. Systolic blood pressure criteria in the National Trauma Triage Protocol for geriatric trauma: 110 is the new 90. J Trauma Acute Care Surg. 2015;78(2):352. 25757122 10.1097/TA.0000000000000523 PMC4620031

[34] Newgard CD, Lin A, Eckstrom E, Caughey A, Malveau S, Griffiths D, et al. Comorbidities, anticoagulants, and geriatric-specific physiology for the field triage of injured older adults. J Trauma Acute Care Surg. 2019;86(5):829. 30629015 10.1097/TA.0000000000002195 PMC6370024

[35] Wasserman EB, Shah MN, Jones CM, Cushman JT, Caterino JM, Bazarian JJ, et al. Identification of a neurologic scale that optimizes EMS detection of older adult traumatic brain injury patients who require transport to a trauma center. Prehosp Emerg Care. 2015;19(2):202–12. 25290953 10.3109/10903127.2014.959225 PMC5070935

[36] Caterino JM, Raubenolt A, Cudnik MT. Modification of Glasgow Coma Scale criteria for injured elders. Acad Emerg Med. 2011;18(10):1014–21. 21951715 10.1111/j.1553-2712.2011.01164.x

[37] Martin JT, Alkhoury F, O’Connor JA, Kyriakides TC, Bonadies JA. ‘Normal’vital signs belie occult hypoperfusion in geriatric trauma patients. Am Surg™. 2010;76(1):65–9. 20135942

[38] Ohio. Health Trauma Network, Ohio Prehospital Trauma Triage Decision Tree—2019 Update. https://ohiohealthems.com/trauma [cited 29.09.2024].

[39] Kim SY, Hong KJ, Do Shin S, Ro YS, Ahn KO, Kim YJ, et al. Validation of the shock index, modified shock index, and age shock index for predicting mortality of geriatric trauma patients in emergency departments. J Korean Med Sci. 2016;31(12):2026–32. 27822945 10.3346/jkms.2016.31.12.2026 PMC5102870

[40] Pandit V, Rhee P, Hashmi A, Kulvatunyou N, Tang A, Khalil M, et al. Shock index predicts mortality in geriatric trauma patients: an analysis of the National Trauma Data Bank. J Trauma Acute Care Surg. 2014;76(4):1111–5. 24662879 10.1097/TA.0000000000000160

[41] Salottolo K, Levy AS, Slone DS, Mains CW, Bar-Or D. The effect of age on Glasgow Coma Scale score in patients with traumatic brain injury. JAMA Surg. 2014;149(7):727–34. 24899145 10.1001/jamasurg.2014.13

[42] Schoeneberg C, Schilling M, Probst T, Lendemans S. Preventable and potentially preventable deaths in severely injured elderly patients: a single-center retrospective data analysis of a German trauma center. World J Surg. 2014;38:3125–32. 25167897 10.1007/s00268-014-2755-0

[43] Kocuvan S, Brilej D, Stropnik D, Lefering R, Komadina R. Evaluation of major trauma in elderly patients—a single trauma center analysis. Wien Klin Wochenschr. 2016. 10.1007/s00508-016-1140-4. 27896468 10.1007/s00508-016-1140-4

[44] Brongel L, Lasek J, Słowiński K. Podstawy współczesnej chirurgii urazowej: Wydawnictwo Medyczne; 2008.

[45] Scheetz LJ, Orazem JP. The influence of sociodemographic factors on trauma center transport for severely injured older adults. Health Serv Res. 2020;55(3):411–8. 31994218 10.1111/1475-6773.13270 PMC7240777

[46] Rozporządzenie. Ministra Zdrowia z 25 stycznia 2016 r. w sprawie centrum urazowego dla dzieci (Dz.U. z 2020 r. poz. 1948).

[47] Calland JF, Ingraham AM, Martin ND, Marshall GT. Geriatric trauma practice 6management guideline (Update) Eastern Association for the Surgery of Trauma. 2017. 10.1097/TA.0b013e318270191f 23114492

[48] Duvall DB, Zhu X, Elliott AC, Wolf SE, Rhodes RL, Paulk ME, et al. Injury severity and comorbidities alone do not predict futility of care after geriatric trauma. J Palliat Med. 2015;18(3):246–50. 25494453 10.1089/jpm.2014.0336 PMC4347887

